# The survival and safety of metastatic hepatocellular carcinoma treated with lenalidomide as second-line therapy: a case report and review of the literature

**DOI:** 10.3389/fonc.2024.1461936

**Published:** 2024-11-20

**Authors:** Tao Li, Ying Zhao, Keren Li, Gong Li, Guangxin Li

**Affiliations:** Department of Radiation Oncology, Beijing Tsinghua Changgeng Hospital, Tsinghua University, Beijing, China

**Keywords:** hepatocellular carcinoma (HCC), lenalidomide, immunomodulatory drug, metastases, case report

## Abstract

**Background:**

Hepatocellular carcinoma (HCC) is a highly lethal and invasive cancer. Targeted and immunotherapies are the primary treatment options for unresectable advanced HCC. There are no recognized and consistent systemic follow-up treatments for patients with HCC who experience disease progression after first-line targeted therapies and immune checkpoint inhibitors (ICIs). According to a few studies, lenalidomide is an immunomodulatory drug that has the potential to be an effective treatment for patients who have progressed after treatment with targeted drugs and ICIs.

**Case summary:**

This article focuses on a patient with HCC whose disease progressed after first-line targeted therapy and ICI therapy combined with lenalidomide as second-line therapy on the basis of the original targeted and ICI regimens, resulting in a favorable oncologic outcome with acceptable toxicity. The progression-free survival (PFS) of the patients in this study reached 3 years, which is much longer than that previously reported, and no progression has occurred thus far.

**Conclusions:**

This case implies that in patients with hepatocellular carcinoma who have failed first-line targeted therapy and ICIs, targeted therapy and ICIs can be restarted with the addition of lenalidomide, with surprising results.

## Introduction

Hepatocellular carcinoma (HCC) is one of the most common cancers in China. Approximately 60-70% of patients have locally advanced or metastatic disease at diagnosis (1). It is a highly lethal and invasive cancer of the liver, and only a small percentage of patients are eligible for potentially curative therapies. Despite the range of localized treatments available to patients, systemic therapy often becomes the treatment of choice for patients who are inoperable and receive other localized treatments. Targeting immune checkpoint inhibitors (ICIs) play an important role in the treatment of HCC and are the main systemic therapeutic option for patients with advanced, metastatic or progressed disease. Recently, several new systemic treatment regimens have been identified from a number of clinical trials for the upfront treatment of advanced and metastatic HCC. However, until recently, there have been no well-recognized and consistent systemic therapies available for patients with HCC who have disease progression on or after first-line targeted therapy and ICIs. Multiple second-line studies are underway, but thus far, there are no definitive results. Some studies have explored new immune checkpoints that show promise as potential new avenues for second-line treatment in liver cancer, such as some emerging phagocytosis checkpoints (2, 3) and bispecific antibodies targeting immunomodulatory checkpoints for cancer therapy (4, 5).

Lenalidomide is an immunomodulatory and antineoplastic agent that was first used for the treatment of multiple myeloma. It is a thalidomide derivative that has similar but more potent activity as an antineoplastic agent. Lenalidomide has immunomodulatory, anti-inflammatory, antiangiogenic and antineoplastic effects. The mechanism of action of these agents in treatment is not well defined but may be related to inhibition of tumor necrosis factor (TNF) alpha, a potent proinflammatory cytokine, or to stimulation of T and NK cell activity (6, 7). Recently, one study revealed that lenalidomide targets the CRL4CRBN ubiquitin ligase to activate the Notch and interleukin-2 pathways in tumor-infiltrating CD8+ T cells. These T cells respond to PD-1-blocking antibodies even in the absence of the CD28 costimulatory receptor after treatment with lenalidomide. Lenalidomide can clinically enhance PD-1 therapy efficacy when treating solid tumors infiltrated with abundant CD28+CD8+ T cells. Molecular glue degraders, such as lenalidomide and pomalidomide, benefit individuals with cancer not only by killing cancer cells directly but also by promoting T-cell-based ICIs (8). We first propose that the efficacy of lenalidomide may be enhanced by combining or following ICIs. Our patient had HCC progression after first-line targeted therapy and ICIs and achieved a better tumor response and longer survival with lenalidomide, and lenalidomide regained efficacy after anti-PD-1 therapy and has not yet progressed.

## Case presentation

### Chief complaints

The patient was a 54-year-old male whose chief complaint was anorexia, and he was subsequently diagnosed with HCC for 3 weeks.

### History of present illness

**Figure 1:**
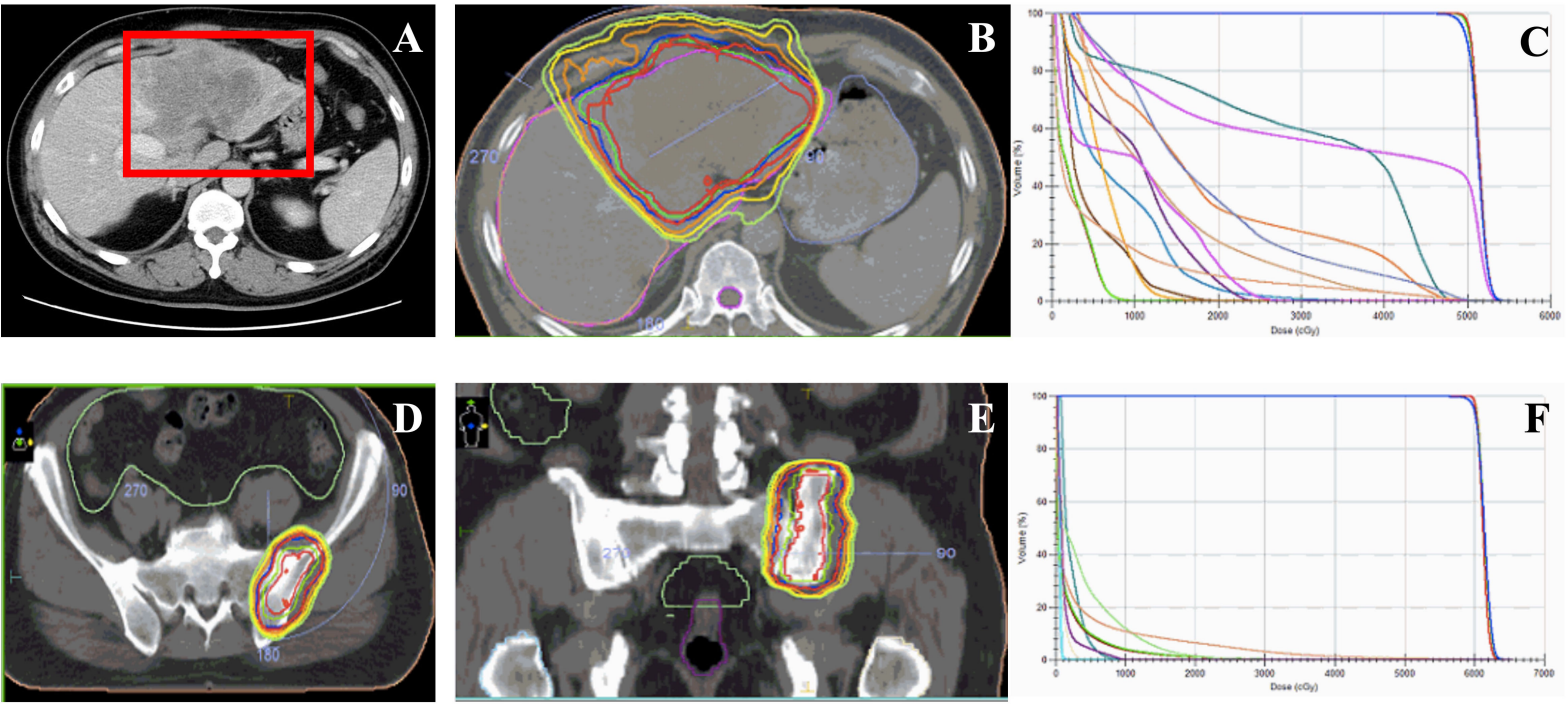
Image of the first patient during the initial treatment and the irradiation treatment plan of the bone metastasis of patient after progressing. **(A)** Liver images of the patient during the course of disease at diagnosis. **(B)** The target volume and the dosimetric distribution of the irradiation of the liver malignant. **(C)** The Dose-Volume Histogram (DVH) of the target volume and organ at risk (OAR) for evaluations of treatment plan. **(D, E)** The target volume of irradiation on the metastasis of the left ilium after three months of lenvatinib treatment. **(F)** The Dose-Volume Histogram (DVH) of the target volume and organ at risk (OAR) for evaluations of treatment plan.

The patient was a 54-year-old man who was diagnosed with HCC on June 7, 2020, with a large mass on his left liver lobe (**Figure 1A**). The levels of alpha-fetoprotein (AFP) and antagonist-II (PIVKA-II) in this patient were significantly greater.

### Personal and family history

No special notes.

### Treatment and efficacy

**Figure 2:**
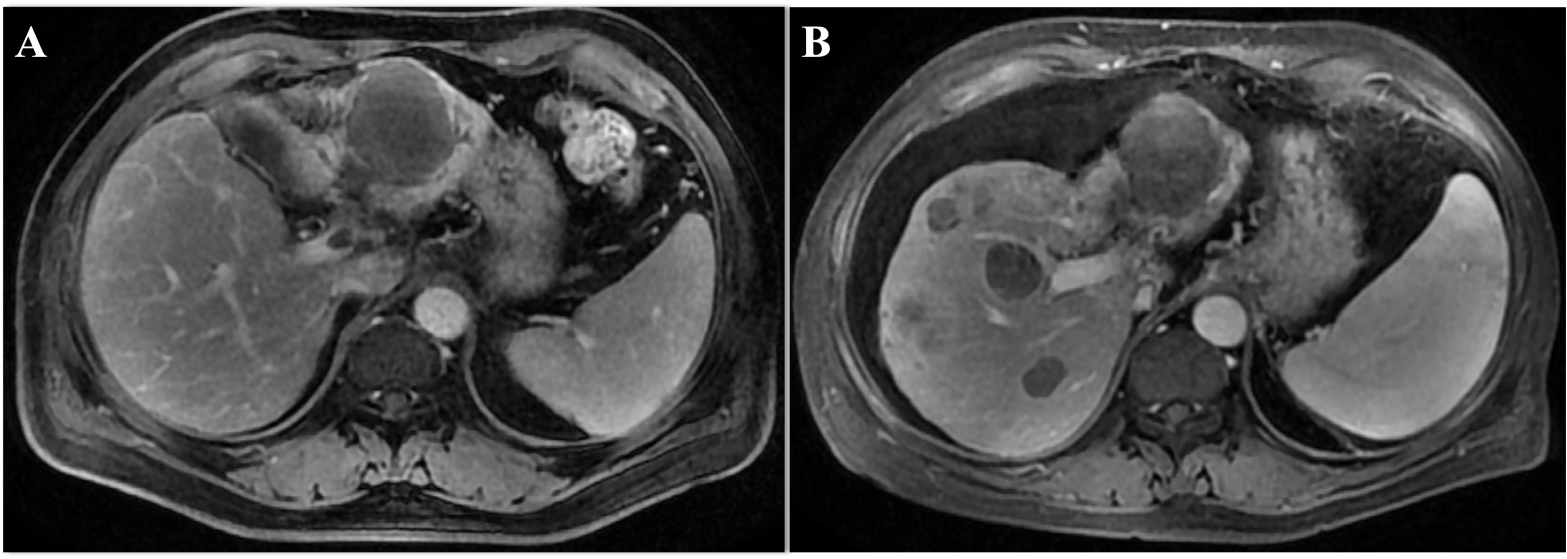
The image of the case during the initial treatment. **(A)** The original tumor size of liver shrinked significantly 1 week after HAIC and anti-PD-L1 treatment. **(B)** The enlarged primary tumor of the left lobe of liver and the new lesion appeared in the right lobe after Lenvatinib and anti-PD-L1 treatment. MRI, magnetic resonance imaging.

**Figure 3:**
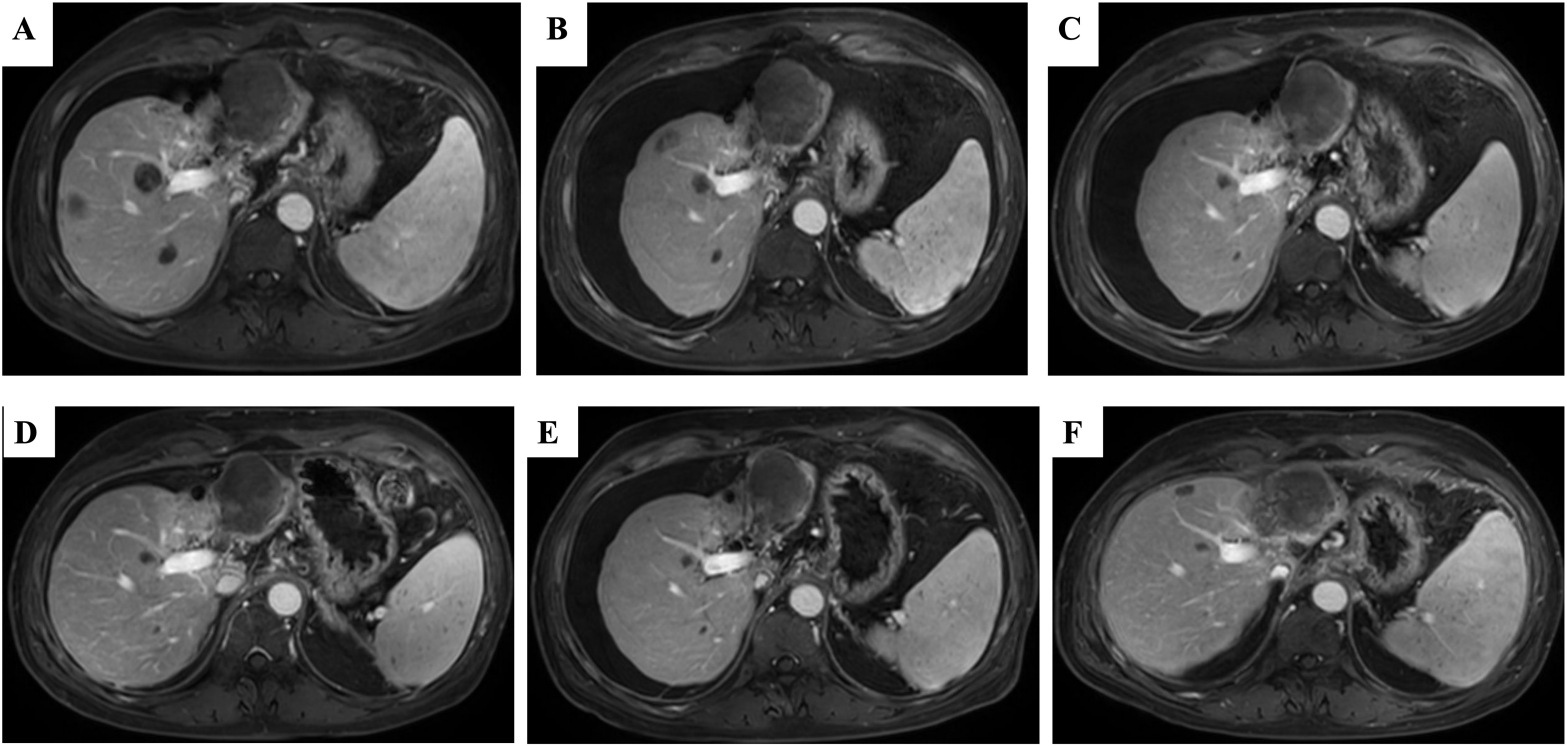
The liver MRI after lenalidomide interruption followed by the initiating of anti-PD-1 treatment and Lenvatinib again. **(A)** The remains of tumor regression after 3 weeks of Camrelizumab plus Lenvatinib. **(B)** The remains of tumor were continuous regressed after 3 months of Camrelizumab plus Lenvatinib. **(C)** The lesions of liver nearly disappeared after 6 months of the combination of lenalidomide and Lenvatinib. **(D–F)** The disease is still responding until now so far. MRI, magnetic resonance imaging.

All ancillary tests revealed that the patient had a portal vein cancerous embolus of the VP3 type at the time of initial diagnosis, and there was no indication for surgery. The patient initially received radiation therapy (RT) targeted to the liver tumor (50 Gy in 25 fractions) from June 25, 2020, to July 7, 2020 (**Figures 1B,C**). Moreover, the patient received lenvatinib (8 mg daily), and the treatment efficacy was evaluated for PR within 3 months until September 2020. Then, a bone scan revealed new metastasis in the left ilium. Radiotherapy was added to the metastasis of the left ilium at 30 Gy in 10 fractions (**Figures 1D–F**) with continuous targeted therapy consisting of lenvatinib and an added anti-PD-1 agent (camrelizumab, 200 mg). On March 15, 2021, hepatic arterial infusion chemotherapy (HAIC) was administered with the FOLFOX regimen because the intrahepatic lesions were still active. Systemic treatment was well tolerated, with a continuous tumor response in the liver for approximately 1 year until June 2021. Magnetic resonance imaging of the liver revealed progression of the liver lesion with new metastases in the right hepatic lobe and enlargement of the original tumor lesion (**Figures 2A, B**). Then, lenalidomide was added on June 25, 2021, accompanied by continuous lenvatinib treatment (8 mg daily) and anti-PD-1 therapy (camrelizumab 200 mg) until now. The patient achieved significant disease control after 3 weeks, 3 months and 6 months of lenvatinib and anti-PD-1 plus lenalidomide treatment (**Figures 3A–C**). Throughout the entire treatment process, the only drug-related adverse reaction observed was a second-degree decrease in the platelet count.

### Outcomes and follow-up

**Figure 4:**
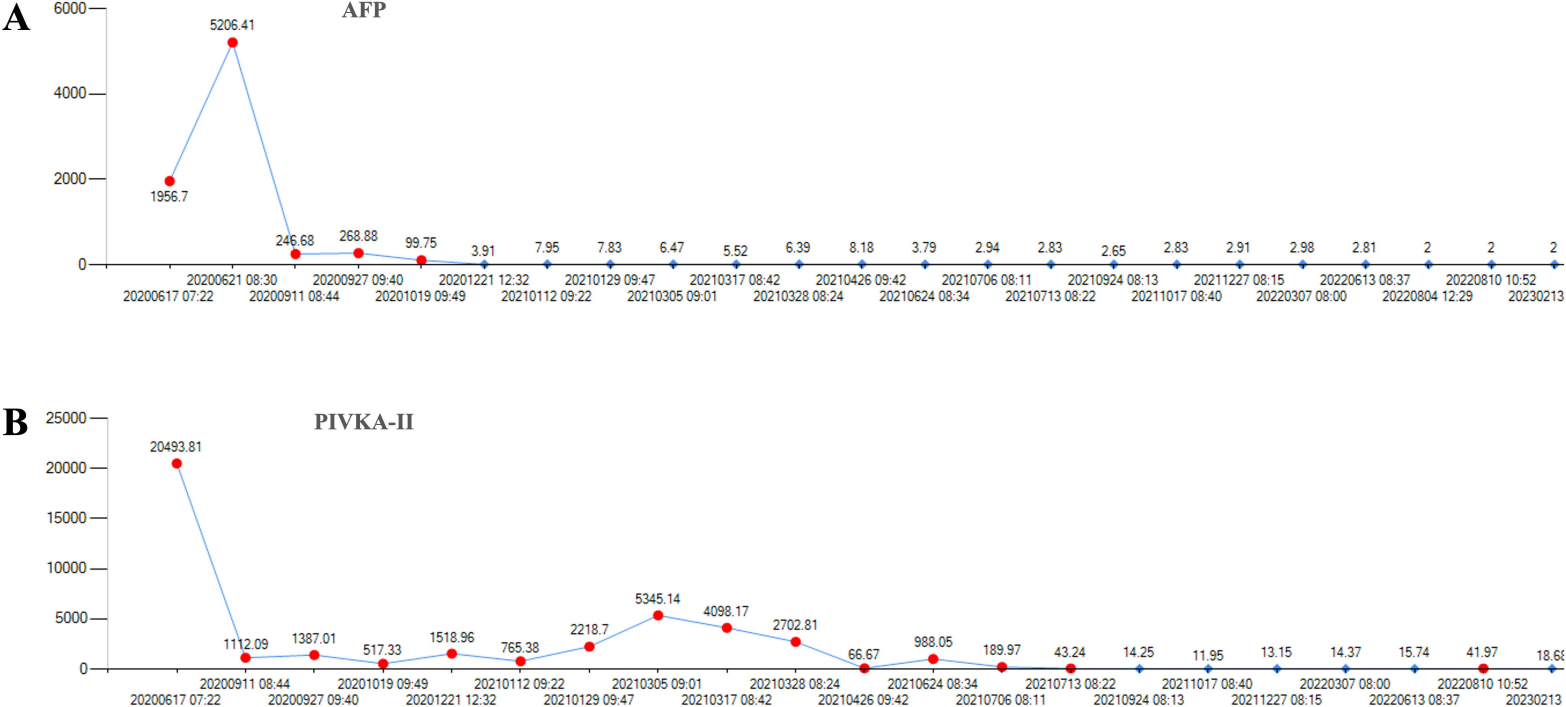
The level of the tumor markers of AFP and PIVKA-II of the first patient during the course of disease. **(A)** There was a continuous decline of AFP after lenalidomide and remained low level until the last follow-up date. **(B)** The level of PIVKA-II was declined after lenalidomide treatment. AFP, alpha-fetoprotein. PIVKA-II, vitamin K absence or antagonist-II.

After 9 months, 1 year and 2 years of treatment with lenalidomide, anti-PD-1 therapy and targeted therapy, the disease still responded (**Figures 3D–F**). The tumor markers AFP and PIVKA-II were continuously at low levels after combination with lenalidomide (**Figures 4A, B**).

In the case report, treatment consent was obtained from the patient, and she is satisfied with the therapeutic schedule and the results so far. We have de-identified the details such that the identity of the patient may not be ascertained in any way. All procedures performed in this study were in accordance with the ethical standards of the institutional and/or national research committee(s) and with the Helsinki Declaration (as revised in 2013). Written informed consent was obtained from the patient for publication of this case report and accompanying images.

## Discussion

There are no subsequent-line systemic therapy options for patients with HCC who experience disease progression after first-line treatment with ICIs. All the clinical trials for second-line treatment are based on sorafenib as the initial treatment. In our case, we presented the disease progression of an HCC patient after ICIs and lenvatinib. After the addition of lenalidomide to ICIs and lenvatinib treatment, the tumor shrunk again and has been controlled for more than 3 years, with no progression to date. At the initial treatment, the patient achieved an approximately 9-month DOR from September 2020 to June 2021 through camrelizumab plus lenvatinib. The progression-free survival (PFS) of the patient was much longer than that previously reported in the IMbrave 150 trial (6.9 months) (9) and the RESCUE trial (5.5 months) (10). A comparison of the combination of lenvatinib and pembrolizumab versus lenvatinib alone as a first-line treatment was completed in a randomized phase III trial, LEAP-002. Although the studies did not meet the primary endpoints (OS and PFS), the ORR was high, and the OS was long; therefore, in practice, this regimen is still used as the first-line treatment option for HCC in many medical centers in China because of its relatively high ORR and reduced risk of gastric bleeding (11). Unfortunately, patients inevitably progress from ICIs and targeted therapy.

Treatment with targeted ICIs is the mainstream first-line treatment for HCC, but there are few clinical data on the optimal treatment after first-line targeted therapy and ICIs. At present, the results of more second-line studies are mainly based on the failure of first-line administration of sorafenib. Therefore, there is an urgent need to find an effective second-line treatment for HCC after the failure of first-line targeted therapy and ICIs.

We considered the immune microenvironment of HCC and the mechanism of action of ICIs. The main inhibitory immune checkpoint receptors that naturally restrain T-cell activity and play vital roles in maintaining self-tolerance include PD-1, CTLA4, LAG3 and TIM3, whereas CD28, GITR and OX40 are examples of costimulatory immune checkpoint proteins that have been shown to enhance T-cell expansion (12). The interaction between PD-L1 and PD-1 leads to broad dephosphorylation of T-cell-activating kinases (13), resulting in T-cell inactivation; thus, PD-1–PD-L1 blockade restores effector CD8+ T-cell function (14). CTLA4 inhibition acts at the T-cell–antigen-presenting cell immune synapse by promoting unopposed interactions of B7 costimulatory ligands with CD28, leading to increased activation of naive CD4+ and CD8+ T cells and rebalancing of the effector and regulatory compartments within the tumor microenvironment (TME) (15, 16). In addition, programmed cell death protein 1 (PD-1) checkpoint blockade therapy requires the CD28 costimulatory receptor for CD8+ T-cell expansion and cytotoxicity. However, CD28 expression is frequently lost in exhausted T cells and during immune senescence, limiting the clinical benefits of PD-1 ICIs in individuals with cancer (17, 18).

Lenalidomide has immunomodulatory, anti-inflammatory, antiangiogenic and antineoplastic effects. Recently, one study using a mouse model of cereblon knockdown showed that lenalidomide reinstates the antitumor activity of CD28-deficient CD8+ T cells after PD-1 blockade. Lenalidomide redirects the CRL4^Crbn^ ubiquitin ligase to degrade Ikzf1 and Ikzf3 in T cells and unleashes paracrine interleukin-2 (IL-2) and intracellular Notch signaling, which collectively bypasses the CD28 requirement for activation of intratumoral CD8+ T cells and inhibition of tumor growth by PD-1 blockade. This research suggested that PD-1 ICIs can benefit from a combination of lenalidomide and solid tumors infiltrated with abundant CD28- T cells (8). Based on these findings, we propose that lenalidomide may be an option when patients develop immune resistance.

In this case, the patient received lenalidomide combination therapy after disease progression following first-line targeted therapy and regained efficacy with ICIs, with tumor control for more than 3 years. The mechanism for this observed activity of ICIs in patients after progression remains unclear but might at least in part be due to the regulation of the TME. The use of lenalidomide alone or in combination with ICIs can allow patients to reobtain efficacy from ICIs. However, there are no data demonstrating the effect of lenalidomide alone or in combination on ICI resistance in HCC patients. This clinical practice provides new insight into the underlying mechanism and clinical trial.

## Conclusions

Our case was the first international study demonstrating that the combination of lenalidomide and ICIs can be very therapeutic for HCC patients who have progressed after first-line therapy, with a low adverse effect profile and a good safety profile. Our case also shows that lenalidomide can increase the efficacy of ICIs, even if the patient is resistant to ICIs at the start of treatment. The novelty of our case is the prolonged use of ICIs in combination with an immunomodulatory drug, which improved survival and delayed resistance to ICIs. This is a case report, and further studies are needed to validate which patients may benefit from lenalidomide. In conclusion, this protocol represents a new therapeutic option for patients with HCC after progression who are receiving targeted immunologic first-line therapy.

## Data Availability

The original contributions presented in the study are included in the article/supplementary material. Further inquiries can be directed to the corresponding authors.
